# The Effect of Ligand Reactivity and Carrier Mechanics on Thiol‐Mediated Cellular Uptake for miRNA Nano‐Drugs

**DOI:** 10.1002/advs.77389

**Published:** 2026-08-27

**Authors:** Zhuang Zhang, Hui Wang, Hao Zhang, Zhenkuan Cao, Zehui Liu, Siying Li, Hongda Wang, Yuping Shan

**Affiliations:** ^1^ College of Chemistry and Life Science Advanced Institute of Materials Science Changchun University of Technology Changchun China; ^2^ State Key Laboratory of Electroanalytical Chemistry Changchun Institute of Applied Chemistry Chinese Academy of Sciences Changchun China

**Keywords:** biophysics, chemistry, endocytosis, ligand, lipid bilayer fusion, lipoic acid, membrane, mesoporous silica, molecular dynamics

## Abstract

Thiol‐mediated uptake (TMU) is a powerful strategy for promoting the cellular uptake of nano‐drugs; however, the mechanochemical principles that coupled ligand reactivity, carrier mechanics, and receptor clustering remain poorly understood. Herein, the effect of carrier rigidity and cyclic disulfide reactivity on the thiol‐mediated cellular uptake of miRNA nano‐drugs was revealed. Compared with lipoic acid (LA), the higher enhancing effect of asparagusic acid (AspA) is identified with the greater affinity, higher binding stability, and more double‐disulfide binding. The greater association of AspA‐TFRC was also visualized by super‐resolution microscopy imaging. Molecular dynamics simulation further verified the superior membrane disruption and deeper insertion induced by AspA. However, both LA and AspA display concentration‐dependent self‐activation and self‐inhibition effects on exchanging with thiols. Furthermore, we provided direct evidence that thiolation can significantly accelerate endocytosis of rigid mesoporous silica nano‐drugs, whereas thiolation will shift the entry cell pathway of soft lipid nano‐drugs from endocytosis to membrane fusion, and AspA is more efficient. This work establishes a mechanochemical framework that links ligand reactivity, carrier mechanics, and receptor engagement to predict the cellular uptake mechanism of a thiol‐mediated nano‐drug delivery system.

## Graphical abstract

The effect of ligand reactivity and carrier mechanics on thiol‐mediated uptake was investigated at the single‐molecule/particle level. The interaction kinetics of LA and AspA with membrane thiols were quantified; AspA is more efficient due to the higher binding affinity, greater binding stability, and more double‐disulfide binding, as well as greater membrane perturbation and deeper insertion capability, which further significantly enhances the cellular uptake efficiency.

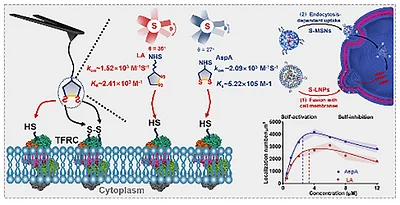

## 1 Introduction

Efficient transmembrane transport of nano‐drugs holds immense promise for treating a wide range of diseases, including infectious diseases, cancers, and various health conditions [1, 2, 3]. In recent years, TMU has emerged as an effective strategy to promote the transmembrane transport of RNA nano‐drugs by exploiting dynamic covalent exchange between cyclic disulfides and exofacial thiols on the cell membrane [4]. Although the TMU has been widely applied, the mechanism by which thiol‐disulfide exchange enhances cellular uptake of nano‐drugs remains insufficiently understood [5, 6]. In particular, it remains unclear how ligand chemical reactivity, carrier mechanics, membrane remodeling, and receptor clustering cooperate to convert molecular membrane anchoring into efficient cellular uptake [7, 8].

Ligand chemical reactivity and carrier mechanics are two determinants regulating interaction kinetics of the cell membrane interface and further cellular uptake dynamics [9, 10]. Despite advances in individual effects of chemical decoration and mechanical stiffness, most studies focus on single modification or uniform carrier rigidity, lacking systematic comparison of sulfur‐based ligands on carriers with disparate stiffness [11]. LA and AspA are widely utilized for TMU delivery systems; however, the discrepancies induced by LA and AspA on the roles of binding affinity, receptor clustering, and membrane perturbation in transmembrane transport remain unclear. Mechanically, soft deformable liposomes (LNPs) and rigid mesoporous silica nanoparticles (MSNs) exhibit distinct membrane responses and internalization preferences upon cellular contact [12]. Studying the kinetics of exchanging with membrane thiols and the dynamics when entry cell at the single‐molecule/particle level will overcome the masking effect of the group average and accurately calculate the dynamic parameters of individual events [13].

Herein, we investigated the kinetics of interacting with the cell membrane and the dynamics of cell entry for miRNA nano‐drugs, which are loaded with LA‐ or AspA‐thiolated soft LNPs and rigid MSNs. The exchange interaction kinetics of thiols from LA and AspA with thiols on the exofacial membrane were quantified at the single‐molecule level. The membrane insertion and lipid disordering induced by LA and AspA were further evaluated by molecular dynamics (MD) simulations. The clustering and association of TFRC on the cell membrane after binding with LA and AspA were visualized at nanoscale using direct Stochastic Optical Reconstruction Microscopy (dSTORM) super‐resolution imaging. Furthermore, the entry cell process of thiolated MSNs nano‐drugs was captured at the single‐particle level by employing AFM‐based force tracing with high temporal resolution, allowing direct comparison of LA‐ and AspA‐mediated cellular uptake dynamics. Meanwhile, the cellular uptake pathway of soft LNPs and rigid MSNs was further identified with direct evidence. This integrated multi‐scale approach enables systematic analysis of the effects of ligand chemical reactivity and carrier mechanics across disparate nanoparticle platforms. This work will suggest validated design principles for guiding the development of TMU‐based nano‐drugs.

## 2 Results and Discussion

### 2.1 Assessment of the Efficiency of LA and AspA in Exchanging Disulfides With Exofacial Thiols

The key step for thiolated miRNA nano‐drugs cellular uptake with high efficiency is the dynamic covalent exchange of disulfides with exofacial thiols on the cell membrane. To precisely elucidate the underlying mechanism by which thiolation enhances miRNA delivery efficiency, the dynamics of LA (a dithiolane‐bearing antioxidant) and AspA (a plant‐derived 1,2‐dithiolane) interacting with thiols on the cell membrane were studied using AFM‐based single‐molecule force spectroscopy (SMFS) under near‐physiological conditions, which allows quantifying the binding/unbinding dynamic features at the single‐molecule level [14]. The NHS group of Fmoc‐NH‐PEG‐NHS was covalently conjugated to the amino‐functionalized AFM tip; the Fmoc‐protected group on the opposite end was removed with 4‐methylpiperidine, and the resulting amino group was coupled to the NHS ester of either LA or AspA (Figure S1). During SMFS measurements, the functionalized AFM tip was approached to the cell membrane, permitting the formation of one or two disulfide bonds between tip‐bound LA/AspA and thiols on the cell membrane (Figure 1A). Then, the AFM tip was retracted to the default position, leading to unbinding of the disulfide bond, and the cantilever deflection was recorded and converted to rupture force, yielding a force‐distance (FD) curve (Figure 1B). The AspA (22.5%) displayed the higher binding probability (BP) with membrane thiols than that of LA (17.8%). After treatment with the thiol‐reducing agent tris(2‐carboxyethyl)phosphine (TCEP), the BP increased to 37.8% and 26.1%, respectively. In contrast, after treatment with the thiol‐blocking reagent N‐ethylmaleimide (NEM), the BP decreased to 6.0% and 4.2%, respectively, suggesting that the density of thiols on the cell membrane modulates the interaction effect (Figure 1C). The rupture force was measured and normalized to a Gaussian function per unit area to generate the probability density function (PDF). The PDF serves as a continuous force histogram, which is fitted with Gaussians, and the peak corresponds to the most probable rupture force; the bimodal profile suggests single‐ and double‐disulfide rupture events. The rupture force for LA is 434 ± 101 pN and 688 ± 232 pN, corresponding to the single‐disulfide rupture and double‐disulfide rupture, whereas the corresponding rupture force for AspA is 603 ± 141 pN and 836 ± 269 pN, respectively (Figure 1D,E). The double‐disulfide accounted for 18% of all rupture events for AspA, higher than that for LA (7.9%). To exclude the influence of other molecules on the complex cell membrane, we performed SMFS analysis on purified TFRC protein immobilized on amine‐functionalized silicon substrates (thickness of approximately 4.1 nm, Figure S2). Single‐ and double‐disulfide rupture events were also observed; the most probable single‐disulfide rupture forces are 426 ± 105 pN and 582 ± 90 pN for LA‐TFRC and AspA‐TFRC, respectively, and the corresponding double‐disulfide rupture forces are 804 ± 248 pN and 996 ± 261 pN (Figure S3A–C). The AspA still displayed a higher BP (7.1%) than that of LA (4.3%, Figure S3D). Moreover, according to the Bell‐Evans two‐state model [15], we described the interaction dynamics between LA/AspA and membrane thiols, in which an applied external force decreased the activation barrier for bond dissociation and thus shortened the bond lifetime (Figure S4). In the far‐from‐equilibrium regime, the rupture force between LA/AspA and membrane thiols increases linearly with the logarithm of the loading rate (LR). We also applied the Williams‐Evans model [16] to account for parallel loading of multiple identical bonds, which is in agreement with the observed high force populations (Figure 1F,G). From the fitted slopes, the separation distance (*X*_β_) was estimated as 0.34 and 0.21 nm for LA and AspA, and the corresponding dissociation kinetic rate constants (*k*_off_) obtained from the intercept at LR = 0 was 6.3 × 10^−1^ s^−1^ and 4.0 × 10^−3^ s^−1^, respectively (Figure 1F,G). The dissociation activation energy (*ΔG*_β,0_) was calculated as 21.19 *k*_B_ *T* and 26.24 *k*_B_ *T* for LA and AspA, respectively. We further estimated the binding‐rate constant (*k*_on_) under the assumption of pseudo‐first‐order kinetics for the receptor‐ligand complex [17]. BP was measured at varying contact time, which increased monoexponentially, yielding interaction times of 290 and 210 ms for LA and AspA, respectively, corresponding *k*_on_ was 1.52 × 10^3^ M^−1^ s^−1^ and 2.09 × 10^3^ M^−1^ s^−1^, respectively (Figure 1H). The equilibrium affinity constant (*K*_a_ = *k*_on_ */k*_off_) indicated weaker affinity of LA (2.41 × 10^3^ M^−1^) to membrane thiols than that of AspA (5.22 × 10^5^ M^−1^). The estimated interaction kinetic parameters summarized in Table S1 indicate that AspA exhibits the higher binding affinity and stability with membrane thiols, which may be attributable to its higher ring tension. It is reported that the interaction efficiency increases with ring tension from LA with *θ* = 35° to AspA with *θ* = 27° [4]. The higher ring tension of AspA not only enhances its thiol‐exchange reactivity but also prolongs its residence time on the membrane through stronger binding affinity and slower dissociation. This powerful dynamic covalent exchange prolongs the membrane accessibility of thiolated nano‐drugs, thereby providing a key driving force for subsequent cellular uptake. Beyond governing binding kinetics, the ring tension of cyclic disulfides may also influence their capacity to perturb and insert into the lipid bilayer [18].

**FIGURE 1:**
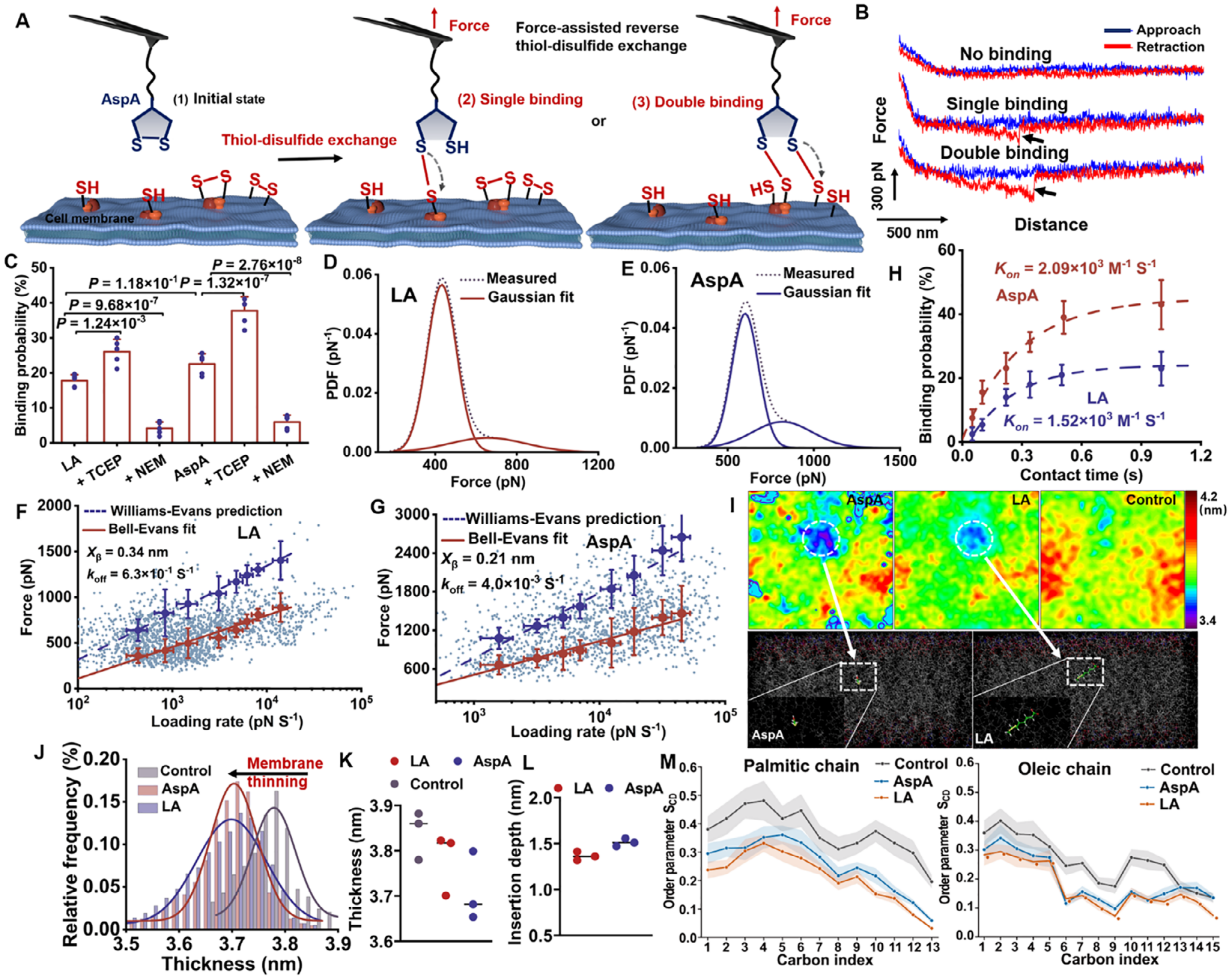
Analysis of the interaction dynamics between LA/AspA and membrane thiols at the single‐molecule level. (A) Schematic diagram of the interaction between AspA on the AFM tip and membrane thiols. (B) Typical FD curves for the interaction between LA and membrane thiols. The black arrows represent the rupture force signal. (C) The BP of LA/AspA binding with membrane thiols before and after treatment with NEM and TCEP. BP was calculated from at least five independent measurement groups. For each group, 3000 randomly selected FD curves were analyzed, and the number of FD curves with a force signal was divided by 3000. (D,E) PDF plots indicating the distribution of rupture force for membrane thiols and LA/AspA; the data were fitted with a bimodal Gaussian model. (F, G) Plots of rupture force vs. loading rate for LA and AspA, respectively. The solid line shows the fit of single‐bond rupture events to the Bell‐Evans equation, yielding *k*_off_ and *X*_β_ values. Dashed lines represent the prediction of the force for the rupture of two simultaneous uncorrelated interactions (Williams‐Evans prediction). (H) BP was plotted as a function of contact time for LA/AspA and membrane thiols. (I) Local membrane thickness heatmap showing spatial thickness distribution for control, LA, and AspA groups. The molecular details for LA and AspA are shown in the bottom panel (the head groups and tail groups of lipid molecules are colored red and white; the carbon, oxygen, and sulfur atoms in LA and AspA are colored green, red, and yellow, respectively). (J) Histogram distribution of membrane thickness for control, LA, and AspA (n = 3). (K,L) Interval plots of membrane thickness and insertion depth for the control, LA, and AspA, respectively. Central dots denote mean values, and the vertical lines represent 95% confidence intervals (n = 3). (M) The S_cd_ of the palmitic chain and oleic chain in lipid molecules (n = 3). The carbon index starts from the carboxyl group of the fatty acid chain. Control indicates that nothing is inserted into the membrane. Data are presented as the mean ± SD. Statistical significance was determined using ordinary one‐way ANOVA with multiple comparisons in H and J (ns, not significant; ^**^ *p*< 0.01; ^****^ *p* < 0.0001).

Effective TMU requires not only stable membrane anchoring but also sufficient penetration into the lipid bilayer to trigger membrane remodeling. Herein, we performed three independent 100 ns all‐atom MD simulations to elucidate the lipid bilayer deformation induced by LA and AspA, reflecting lipid reorganization and insertion of cyclic disulfides. Heatmaps of membrane thickness revealed localized thinning at insertion loci, with LA producing modest thinning and AspA inducing more pronounced thinning and curvature in the insertion region compared with the control group (Figure 1I,J). The AspA likely provides greater driving force for perturbing adjacent lipids, leading to increased local disruption and deeper penetration into the bilayer (Figure 1K,L). We further quantified membrane order by analyzing the deuterium order parameter (S_cd_), which quantifies lipid tail ordering with S_cd_ = 0 corresponding to isotropic orientation and S_cd_ = 1 corresponding to orthogonal alignment to the bilayer plane [19, 20]. In the presence of AspA, substantial decreases in |S_cd_| values were observed in the mid‐to‐terminal regions of the lipid tails, indicating increased chain disorder and reduced stacking order (Figure 1M). In addition, we calculated the insertion potentials of mean force (PMF) using umbrella sampling with 36 sampling windows and 200 bootstrap profiles. Relative to the bulk aqueous reference, AspA exhibited a deeper PMF minimum at approximately 0.8 nm, whereas LA showed a shallower minimum at approximately 1.5 nm (Figure S5). These results indicate that AspA achieves greater membrane insertion depth and more extensive lipid disordering, which facilitates bilayer reorganization. The efficiency of TMU also depends on the organization of receptor clustering bound with ligands across the nanometer‐to‐micrometer scale on the live cell membrane.

### 2.2 Visually Quantitative Analysis of LA/AspA Exchanging Disulfides With Membrane Thiols

We further employed dSTORM imaging to visually quantify the receptor clustering when LA/AspA exchanged disulfides with membrane thiols, which enables subdiffraction‐resolution visualization [21]. Herein, LA‐Cy5 and AspA‐Cy5 were successfully obtained by amidation and click chemistry (Figure S6). Before performing dSTORM imaging, the LA‐Cy5 or AspA‐Cy5 was incubated with HeLa cells for 10 min at different concentrations (0.5–12 µM) (Figure 2A–C and Figure S7). The localization density (LD, localizations µm^−2^) for AspA was 821, 2101, 3364, 4191, 3777, 3404, and 2814 at 0.5, 1, 2, 4, 6, 8, and 12 µM, respectively. The corresponding LD for LA was 421, 1512, 2224, 2710, 3114, 2267, and 1812, respectively. It was found that under low labeling concentration (<4 µM), LD increased with increasing probe concentration, and the clusters became more intact and well defined. However, when the concentration was higher than 6 µM, the LD for both probes decreased as the probe concentration increased (Figure 2D), which may be attributed to two competing processes. At low to moderate concentration, binding of AspA or LA to membrane thiols promotes local enrichment of thiol‐bearing species and cluster growth, consistent with a self‐activation process. At higher concentration, LA or AspA can undergo intermolecular thiol‐disulfide exchange to form dense surface‐adsorbed layers or micelles, which likely sterically impede contact with membrane thiols and thus trigger a self‐inhibitory effect. To capture these opposing regimes quantitatively, LD as a function of concentration was fitted according to the biphasic Hill model [22]; the half‐maximal effective concentration (EC_50_) for AspA was 1.2 µM compared with 2.7 µM for LA, whereas the half‐maximal inhibitory concentration (IC_50_) for AspA was 10.0 vs. 12.8 µM for LA (Figure 2D), AspA exhibited a lower activation threshold and an earlier onset of inhibition relative to LA. Flow cytometry analysis of the cells labeled with FITC‐LA and FITC‐AspA also revealed dose‐dependent self‐activation at low concentration and self‐inhibition at high concentration (Figure 2E). This biphasic behavior suggests that at low density, clustered ligands cooperatively stabilize membrane binding, whereas at excessively high density, steric crowding or thiol depletion at the membrane surface limits further binding, thereby imposing an inherent upper bound on TMU efficiency. Notably, AspA reached its self‐inhibition threshold at a higher localization density than LA, indicating a wider productive concentration window for TMU. We also analyzed the distribution of cluster diameter using the SR‐Tesseler method [23]; both LA and AspA produced progressively larger clusters with increasing labeling concentration, and the cluster diameters were predominantly distributed in the range of 250–350 nm at a concentration of 12 µM (Figure 2F), and the cluster size was modulated by the density of membrane thiols (Figure 2G). Meanwhile, the LD and cluster number per µm^2^ for AspA were also larger than those for LA even after treatment with NEM and TCEP, respectively (Figure 2H). For each dataset, the plot of the localization number per cluster (N_C_) vs. cluster area (C_A_) was fitted to a linear equation [24], where the slope 𝜌_C_ is the clustering density in the limit of zero area. AspA exhibited a higher 𝜌_C_ (1.12 × 10^−2^ loc nm^−2^) than that of LA (0.95 × 10^−2^ loc nm^−2^, Figure 2I), indicating that membrane thiols bound with AspA form denser and more cooperative clusters. After TCEP pretreatment, both LA and AspA exhibited increased N_C_ and C_A_ values, attributed to enhanced thiol density; conversely, NEM pretreatment resulted in significantly reduced N_C_ and C_A_ values. These properties are expected to affect the cellular uptake pathway and efficiency of TMU‐based nano‐drugs, which is modulated by the ligand and nanocarrier of nano‐drugs.

**FIGURE 2:**
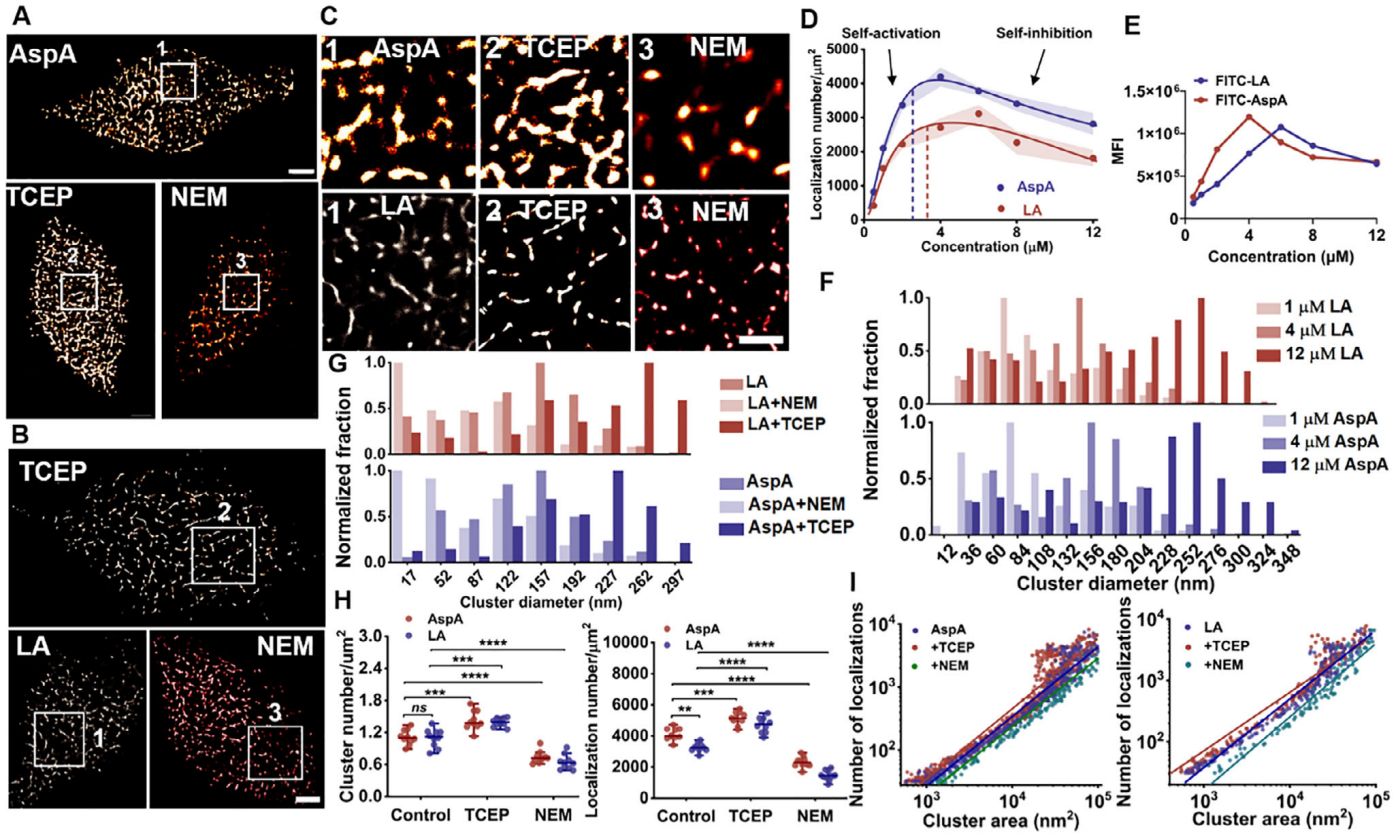
Quantitative analysis of the spatial distribution of LA/AspA binding with membrane thiols on HeLa cells. (A,B) Representative dSTORM images of membrane thiols bound with (A) AspA and (B) LA before and after NEM and TCEP treatment. Scale bar = 4 µm. (C) Images of enlarged regions 1, 2, and 3 from Figures A and B (white boxes). Scale bar = 1 µm. (D) LD after binding with AspA and LA at different concentrations; the shadow around the curve represents the standard error band. The solid lines are the corresponding biphasic Hill fits (n = 10). (E) MFI of cells after binding with AspA and LA at different concentrations. (F) Normalized distribution of cluster diameters in HeLa cells labeled with AspA and LA at varying concentrations. (G) Normalized distribution of cluster diameters for AspA‐ and LA‐labeled membrane thiols before and after treatment with TCEP or NEM. (H) The cluster number per µm^2^ and LD of AspA and LA binding to membrane thiols at 4 µM before and after pre‐incubation with TCEP or NEM (n = 10). (I) Plot of N_C_ vs. C_A_ for AspA/LA (blue), TCEP (red), and NEM (green) datasets; linear fits were used to derive 𝜌_C_. Data are presented as the mean ± SD. Statistical significance was determined using ordinary one‐way ANOVA with multiple comparisons (ns, not significant; ^**^ *p* < 0.01; ^***^ *p* < 0.001; ^****^ *p* < 0.0001).

### 2.3 Preparation and Characterization of Soft and Rigid Thiolated miRNA Nano‐Drugs

To evaluate the cellular uptake dynamics of nano‐drugs that are composed of LA‐ or AspA‐modified nano‐carriers with contrasting mechanical properties, we selected two mechanically distinct platforms, soft LNPs and rigid MSNs thiolated with cyclic disulfide LA and AspA, respectively. LA and AspA were independently conjugated to the terminal amine of DSPE‐PEG‐NH_2_ via amide reaction, yielding thiolation precursors SPD‐1 and SPD‐2, respectively (Figure 3A), which were confirmed by ^1^H NMR spectroscopy and energy dispersive spectroscopy (Figures S8–S11). The yields, determined by the ninhydrin assay [25], were quantified as 61.7% and 67.8% for SPD‐1 and SPD‐2, respectively (Figure S12). Subsequently, the SPD‐1 and SPD‐2 were separately incorporated into a conventional LNP formulation containing SM‐102, DSPC, DSPE‐PEG, and cholesterol using the thin‐film hydration method [26], yielding disulfide‐functionalized LNPs denoted LA‐LNPs and AspA‐LNPs, respectively (Figure 3B). To evaluate the effect of thiolated lipid content on LNP cellular uptake, we prepared three variants with different molar ratios of SPD‐1 or SPD‐2, designated LA‐LNP1, LA‐LNP2, LA‐LNP3, and AspA‐LNP1, AspA‐LNP2, AspA‐LNP3 (Figure S13 and Table S2), respectively. It is found that thiolated LNPs consistently promote cellular uptake compared with conventional LNPs; notably, LA‐LNP2 (DSPE‐PEG: SPD‐1 = 0.8:0.7, molar ratio) and AspA‐LNP2 (DSPE‐PEG: SPD‐2 = 0.8:0.7, molar ratio) exhibit the highest enhancements, with cellular uptake events increasing 2.56‐fold and 3.01‐fold relative to LNPs, respectively (Figure 3C). Then, a model miRNA (micrON mimic NC) was encapsulated into LA‐LNP2 and AspA‐LNP2 for subsequent experiments, showing a high encapsulation efficiency (>70%) quantified using the RiboGreen fluorescence assay (Figure 3D and Figure S14). The resulting LNPs exhibited uniform diameter with narrow dispersity (PDI< 0.2) and negative zeta potential owing to the contribution of encapsulated negatively charged miRNA and anionic surface moieties (Figure 3E). Meanwhile, the thiolated LNPs maintained excellent size stability during prolonged storage and exhibited low cytotoxicity, supporting their suitability as nano‐drugs (Figure 3F and Figure S15). MSNs, widely used inorganic biomaterials for nano‐drug carriers, were selected as the rigid carrier to study the cellular uptake mechanism of thiolated nanoparticles. LA and AspA were conjugated to the surface of commercial MSNs (with a diameter of 120 nm, positive zeta potential, and low cytotoxicity) via an amide reaction to obtain LA‐MSNs and AspA‐MSNs. Successful surface thiolation was confirmed by Fourier‐transform infrared spectroscopy (Figure S16). Then, miRNA was encapsulated within the pore network of the thiolated MSNs (Figure 3B); the encapsulation efficiency of MSNs is lower than that of LNPs, especially after thiolation with LA and AspA, which may be attributed to the steric effects of LA and AspA on the surface of rigid MSNs. dSTORM images demonstrated the good monodispersity, and AFM imaging confirmed the spherical morphology (Figure 3G and Figures S17–S19). AFM‐based nanoindentation measurements revealed that the Young's modulus of MSNs (73.88 MPa) is three orders of magnitude higher than that of LNPs (0.07 MPa), reflecting a pronounced disparity in mechanical stiffness, and the thiolation did not induce significant changes in particle stiffness (Figure 3H).

**FIGURE 3:**
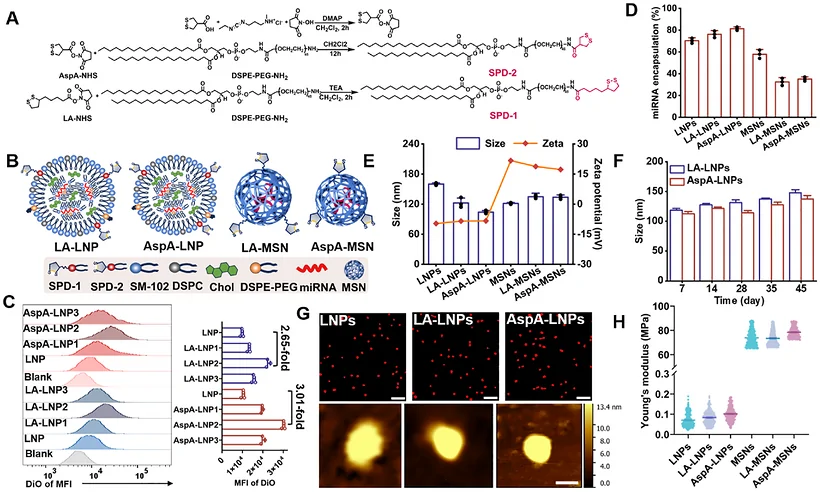
Preparation and characterization of thiolated LNPs and MSNs. (A) Synthetic schemes for SPD‐1 and SPD‐2. (B) Schematic diagram of the thiolated miRNA nano‐drugs. (C) Flow cytometry analysis of HeLa cells incubated with DiO‐labeled LNPs with different contents of cyclic disulfides (n = 5). (D) Encapsulation efficiency of miRNA in LNPs and MSNs (n = 3). (E) Hydrodynamic diameter and zeta potential of LNPs and MSNs (n = 3). (F) Stability of miRNA‐loaded LA‐LNPs and AspA‐LNPs over time during storage at 4°C (n = 3). (G) Representative dSTORM (top panel, scale bar = 1 µm) and AFM (bottom panel, scale bar = 100 nm) images of miRNA‐loaded LNPs, LA‐LNPs, and AspA‐LNPs. (H) Young's modulus of miRNA‐loaded LNPs and MSNs; data are presented as mean ± SD (n = 400).

### 2.4 Study the Effect of Thiolation on the Cellular Uptake of Rigid and Soft miRNA Nano‐Drugs

As a well‐established inorganic nano‐carrier platform for RNA delivery [27], MSNs were selected as the model of rigid nanocarrier to examine the cellular uptake behavior. The MSNs were thiolated via amide coupling to yield LA‐MSNs and AspA‐MSNs, which were subsequently labeled with Cy5 for fluorescence analysis. Flow cytometry analysis revealed that the MFI of Cy5 from LA‐MSNs and AspA‐MSNs increased 2.5‐fold and 5.3‐fold, respectively, compared to unmodified MSNs (Figure 4A). After pretreatment with TCEP, the relative cellular uptake rate for LA‐MSNs and AspA‐MSNs increased by 82.6% and 42.1%, respectively. After pretreating the cells with NEM, the corresponding cellular uptake rate was reduced by 89.2% and 81.2%, respectively. No significant change in cellular uptake rate was observed for unmodified MSNs after the above reagent pretreatments (Figure 4B,C). To further study the internalization mechanism, CPZ, Filipin III, and EIPA were used as pharmacological inhibitors of clathrin‐mediated endocytosis, cholesterol‐dependent lipid raft‐associated uptake, and macropinocytosis, respectively [28]. Low‐temperature incubation and treatment with CPZ or Filipin III produced formulation‐dependent changes in MSN uptake (Figure 4B,C). These results suggest that temperature‐sensitive processes, clathrin‐associated uptake, and cholesterol‐rich membrane domains may contribute to the cellular internalization of the MSNs. Furthermore, CLSM imaging was used to examine the cellular distribution of the MSNs; the MSNs were labeled with Cy5 (red), while the HeLa cell membrane was labeled with DiO (green). After 2 h of incubation, the Cy5‐labeled MSNs exhibited predominantly intracellular and perinuclear accumulation with limited overlap with the plasma membrane signal (Figure 4D).

**FIGURE 4:**
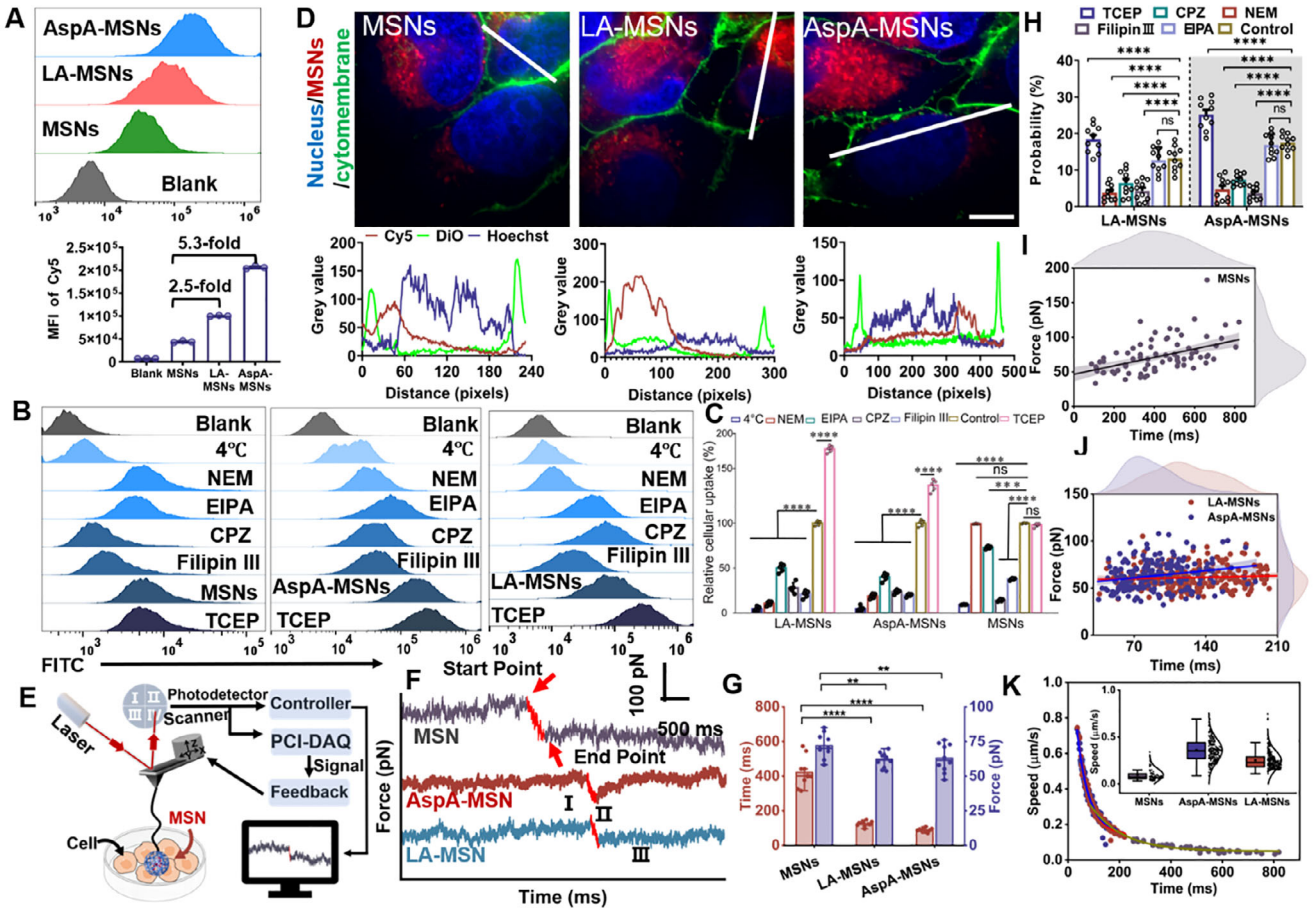
Evaluating the cellular uptake mechanism of thiolated MSNs. (A) Flow cytometry analysis of HeLa cells incubated with Cy5‐labeled MSNs, LA‐MSNs, and AspA‐MSNs for 1 h (n = 3). (B) Representative flow cytometry plots of HeLa cells pretreated under different conditions, followed by co‐incubation with MSNs, LA‐MSNs, or AspA‐MSNs for 2 h. (C) Quantification of the relative cellular uptake rate of MSNs under the indicated treatment. The control group denotes the corresponding MSNs, LA‐MSNs, and AspA‐MSNs groups, respectively; the relative cellular uptake rate of the control group is normalized to 100%. (D) CLSM images showing DiO‐labeled cell membrane (green), Cy5‐labeled MSNs (red), and nuclei (blue). Scale bar = 10 µm. (E) Schematic diagram of the force tracing workflow. (F) The typical force tracing curve for a single MSN entry cell; red segments indicate the entry cell process. (G) Histogram distribution of force and duration during MSN entry cell (n = 75), LA‐MSNs (n = 298), and AspA‐MSNs (n = 335). (H) Probability of observing FT signal before (Control denotes LA‐MSNs or AspA‐MSNs) and after treatment with NEM, TCEP, CPZ, EIPA, and Filipin III. (I,J) Distribution of the uptake force as a function of the duration for MSN, LA‐MSN, and AspA‐MSN. The marginal plots show the normal distribution of force and time. Linear regression (solid line) was used to fit the force‐time trend, and the Pearson correlation analysis was employed to assess the relationship between time and force. (K) Distribution of the uptake speed as a function of the duration for MSN, LA‐MSN, and AspA‐MSN. Nonlinear regression (solid line) was used to simulate the relationship between speed and time. Data are presented as the mean ± SD. Statistical significance was determined using ordinary one‐way ANOVA with multiple comparisons (ns, not significant; ^**^ *p* < 0.01; ^***^ *p* < 0.001; ^****^ *p* < 0.0001).

Then, we explored the endocytosis dynamic mechanism of MSNs using the force tracing technique at the single‐particle level, which could detect the fast entry cell behavior of a single nanoparticle with a time resolution of 50 µs and a force resolution of 10 pN [13]. LA‐MSNs and AspA‐MSNs were connected to the ammoniated AFM tip through the Acetal‐PEG‐NHS linker, respectively, in which the NHS group anchors to the AFM tip and the aldehyde terminus covalently reacts with the amino group on MSNs (Figure S20). The principle of the force tracing technique is shown in Figure 4E. Before conducting the force tracing measurement, a CCD camera was used to help locate the functionalized AFM tip on the cell membrane (Figure S21). The tip was then approached toward the cell surface with a velocity of 0.1 µm s^−1^, and then the force‐distance (FD) curves were acquired to determine the exact contact point between the AFM tip and the cell membrane (Figure S22). Finally, the feedback system of the AFM setup was turned off, and a PCI data‐acquisition system was used to record the deflection of the AFM tip cantilever. During force tracing measurements, when LA/AspA on the MSN was recognized, the cyclic disulfide would exchange disulfide with thiols on the cell membrane (Figure S23, stage I), inducing cellular uptake (Figure S23, stage II). During cellular uptake, the AFM tip cantilever will be deflected, which is reflected to the photodetector via the laser beam. Over time, this deflection was recorded and converted into a force‐time (FT) curve by the PCI data acquisition module; the typical FT curves for a single MSN entry cell are shown in Figure 4F. Usually, we recorded around 2000 FT curves on 10 cells for each group, and the number of FT curves with a force signal was divided by 2000 to obtain the percentage of obtaining the force signal. At least three independent experimental groups were performed under each condition. The percentage was calculated as 4.3%, 13.1%, and 17.5% for MSNs, LA‐MSNs, and AspA‐MSNs, respectively (Figure 4H and Figure S24). Pretreating the cells with NEM decreased the probability to 3.8% and 4.7% for LA‐MSNs and AspA‐MSNs, respectively, whereas TCEP pretreatment increased the probability to 18.4% and 25.2%. Meanwhile, inhibitors of clathrin‐ and caveolin‐mediated endocytosis markedly reduced the uptake rate of thiolated MSNs (Figure 4H). It is concluded that AspA‐MSNs and LA‐MSNs are predominantly internalized through receptor‐mediated endocytosis.

The receptor‐mediated endocytosis dynamic parameters were further analyzed; force and duration for a single MSN entry cell were extracted from the FT signal. The distance along the *x* and *y* axes between the start point and the end point represents the duration and force, respectively. The baseline noise level is 7.7 pN, with a signal‐to‐noise ratio of 16.5 dB (Figure S25). The cellular uptake force for LA‐MSNs (61 ± 11 pN) and AspA‐MSNs (63 ± 15 pN) is slightly smaller than that for unmodified MSNs (71 ± 8 pN, Figure 4G). However, the average duration for MSNs (412 ± 166 ms) is considerably longer than that for LA‐MSNs (128 ± 39 ms) and AspA‐MSNs (90 ± 31 ms); thiolation significantly reduced the duration of MSNs' cellular uptake. Furthermore, correlation analysis showed that the uptake force is positively associated with duration (Figure 4I,J). Meanwhile, the displacement during the entry cell process was calculated (Figure S26), and the average speed (displacement/duration) was calculated as 0.09, 0.25, and 0.36 µm s^−1^ for MSNs, LA‐MSNs, and AspA‐MSNs, respectively (Figure 4K). It was found that thiolation significantly accelerates MSN internalization, and AspA is more efficient.

During receptor‐mediated endocytosis, TFRC was evaluated as a candidate mediator, and a covalent bond between AspA and cysteines formed at the positions of 556 and 558 on the surface of the TFRC, resulting in efficient cellular uptake [4]. Herein, TFRC was labeled with Cy3‐T7, while AspA, LA, AspA‐MSNs, and LA‐MSNs were labeled with Cy5. Dual‐color dSTORM imaging was employed to map the spatial relationships between LA/AspA and TFRC on the cell membrane. Representative full‐field and magnified reconstruction images revealed the clear colocalization of AspA, LA, AspA‐MSNs, and LA‐MSNs with TFRC on the HeLa cell membrane (Figure 5A). To quantify the association, we calculated the relative enrichment (RE) via Voronoi tessellation and classified the localizations by nearest‐neighbor distance (NND, nm). RE >1 denotes enrichment of the probe near the reference localizations [29]. The average RE for AspA, LA, AspA‐MSNs, and LA‐MSNs was quantified in zoomed regions centered on TFRC reference sites to evaluate the probe enrichment at defined spatial regions. All probes exhibited enrichment at TFRC within a 10 nm spatial scale, indicating nanoscale colocalization (Figure 5B,D). Coordinate‐based colocalization (CBC) analysis [30] demonstrated that AspA and AspA‐MSN localizations were more frequently represented in the high CBC score range (0.7–1.0) than those of LA and LA‐MSN (Figure 5C,D). These results demonstrate the preferential association of AspA probes with TFRC‐enriched membrane nanodomains. However, spatial colocalization alone does not establish direct molecular binding or a functional role for TFRC [31]. To determine whether TFRC functionally contributes to nanocarrier uptake, TFRC expression was silenced using siRNA, and cellular internalization of MSNs, LA‐MSNs, and AspA‐MSNs was analyzed by flow cytometry. TFRC expression was reduced to 43.2% of control. TFRC silencing reduced the uptake of unmodified MSNs by only 5.1%, but significantly decreased the uptake of LA‐MSNs and AspA‐MSNs by 66.2% and 48.8%, respectively (Figure S27). Collectively, the dSTORM and TFRC loss‐of‐function results suggest that TFRC is a functional contributor to enhance the cellular uptake of LA‐MSNs and AspA‐MSNs.

**FIGURE 5:**
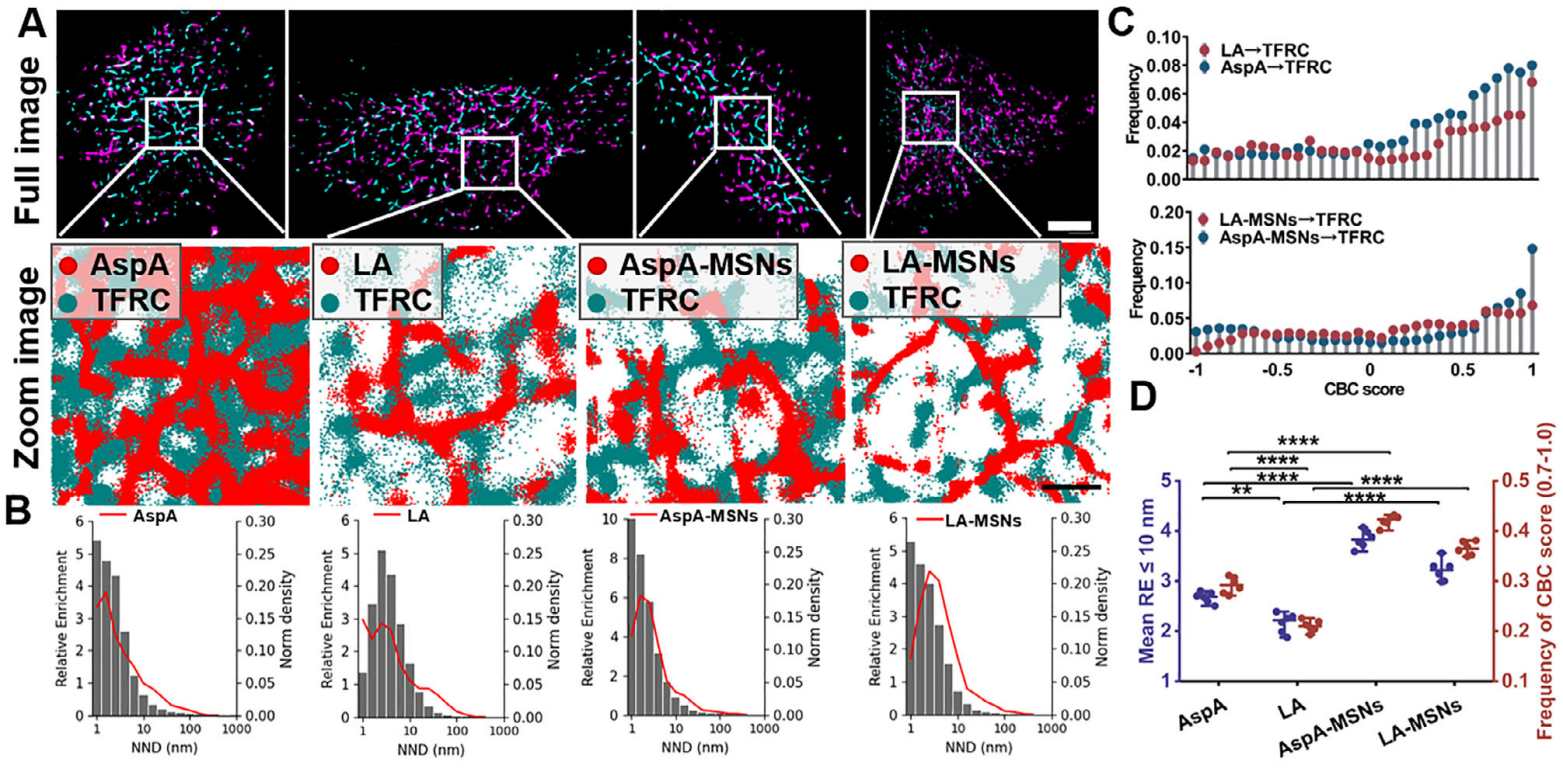
Investigating the potential target receptor for LA and AspA. (A) Representative full‐view and zoomed‐in merged dSTORM images showing colocalization of AspA, LA, AspA‐MSNs, and LA‐MSNs with TFRC, respectively. Scale bar is 4 µm (top) and 1 µm (bottom), respectively. (B) Mean RE density score across AspA, LA, AspA‐MSNs, and LA‐MSNs. Reference regions are binned by NND, and the mean RE density score is calculated for each bin. The RE density score is plotted on the left‐hand *y*‐axis, and relative bin distribution is plotted on the right‐hand *y*‐axis. (C) Histogram distribution of colocalized CBC values for TFRC and AspA, LA, AspA‐MSNs, and LA‐MSNs. The higher the CBC value (approaching 1), the greater the degree of colocalization. (D) Mean RE of TFRC‐enriched regions with an NND ≤ 10 nm for AspA, LA, AspA‐MSNs, and LA‐MSNs (blue), and the summation of frequency in each CBC‐score bin between 0.7 and 1.0 (red). Statistical data were obtained from 10 cells in three independent experiments. Data are presented as the mean ± SD. Statistical significance was determined using ordinary one‐way ANOVA with multiple comparisons (^**^ *p* < 0.01; ^****^ *p* < 0.0001).

To assess the effect of thiolation on the cellular uptake of soft LNPs, the cellular uptake pathway and efficiency were measured using flow cytometry. After co‐incubating HeLa cells with DiO‐labeled LNPs for 1 h, the mean fluorescence intensity (MFI) of DiO from LA‐LNPs and AspA‐LNPs increased 2.9‐fold and 4.0‐fold relative to unmodified LNPs, respectively (Figure 6A). The enhancement is attributed to the disulfide exchange between LA/AspA and the membrane thiols [32]. After pretreating the cells with TCEP, the cellular uptake rate for LA‐LNPs and AspA‐LNPs increased by 61.6% and 42.0%, respectively. In contrast, pretreatment of the cells with the NEM reduced the uptake rate of LA‐LNPs and AspA‐LNPs by 56.6% and 70.2%, respectively, the density of thiols on the cell membrane modulates the TMU effect (Figure S28). By contrast, the above pretreatments did not alter the cellular uptake rate of unmodified LNPs (Figure 6B,C). To dissect the cellular uptake pathway, HeLa cells were pretreated with a panel of pathway‐selective inhibitors. It was found that the cellular uptake of LA‐LNPs and AspA‐LNPs was unaffected by 4°C treatment, and the inhibitors of clathrin‐mediated endocytosis, caveolin‐mediated endocytosis, and macropinocytosis did not exert an effect. However, after pretreating the cells at 4°C or with CPZ, the cellular uptake rate of unmodified LNPs was significantly reduced to 14.4% and 7.5%, respectively, indicating that the cellular uptake of unmodified LNPs is energy‐dependent and mainly dependent on clathrin‐mediated endocytosis (Figure 6B,C). We hypothesized that LA‐LNPs and AspA‐LNPs enter HeLa cells via fusion with the plasma membrane. The pathway of thiolated LNPs entry cell was further confirmed using confocal laser scanning microscopy (CLSM) imaging. LNPs were labeled with DiI (red), and the HeLa cell membrane was labeled with DiO (green). After co‐incubation with cells for 2 h, the red fluorescence from LA‐LNPs or AspA‐LNPs nearly overlapped with the green fluorescence from the cell membrane, and the gray value curve showed clearer overlapping, indicating that LA‐LNPs and AspA‐LNPs could fuse with cell membranes. However, no significant colocalization of green and red fluorescence was observed in the LNPs group (Figure 6D). Additionally, we conducted a fluorescence resonance energy transfer assay to simulate the fusion of thiolated LNPs with the plasma membrane according to the reported methods [33]. Thiolated LNPs labeled with the donor dye DiO (excitation/emission: 488/501 nm) and the acceptor dye DiI (excitation/emission: 553/565 nm) were co‐incubated with HeLa cells. The decrease in DiI fluorescence intensity, accompanied by an increase in DiO fluorescence intensity, indicated the occurrence of membrane fusion, lipids labeled with DiO and DiI transferring from thiolated LNPs to the cell membrane (Figure 6E,F). After a long co‐incubation time (90 min), AspA‐LNPs obtained greater fusion efficiency (57.9%) than that of LA‐LNPs (39.1%), as shown in Figure 6G and Figure S29. To assess whether lipid mixing was accompanied by cargo release, DiD‐labeled LNPs carrying Cy3‐miRNA were analyzed by confocal microscopy. In cells treated with unmodified LNPs, DiD and Cy3 signals were highly colocalized within intracellular puncta, indicating that the cargo was associated with the lipid carrier. In contrast, for LA‐LNPs and AspA‐LNPs, DiD signals were mainly localized at or near the plasma membrane, while Cy3‐miRNA signals were distributed inside the cells (Figure S30), which supports intracellular cargo release and suggests that the FRET signal is not solely due to surface accumulation of intact thiolated LNPs. To further investigate membrane perturbation induced by thiolated LNPs, fluorescence recovery after photobleaching (FRAP) measurements were performed on thiol‐containing supported lipid bilayers (SLBs) to evaluate their effects on lateral lipid diffusion and membrane fluidity. Compared with the untreated control, LA‐LNPs reduced the effective diffusion coefficient and mobile fraction by 71.3% and 64.1%, respectively, whereas AspA‐LNPs produced reductions of 82.7% and 77.2%, respectively. These results indicate that cyclic disulfide functionalization restricts lateral lipid mobility, with AspA‐LNPs producing the strongest membrane‐dynamic perturbation (Figure S31). These results demonstrate that the incorporation of dithiolane shifts the uptake pathway of soft LNPs from endocytosis to membrane fusion, enhancing cellular uptake efficiency, especially for AspA. Thus, across two mechanically disparate nano‐carrier platforms, thiolation exerts mechanistically distinct but functionally enhancing effects.

**FIGURE 6:**
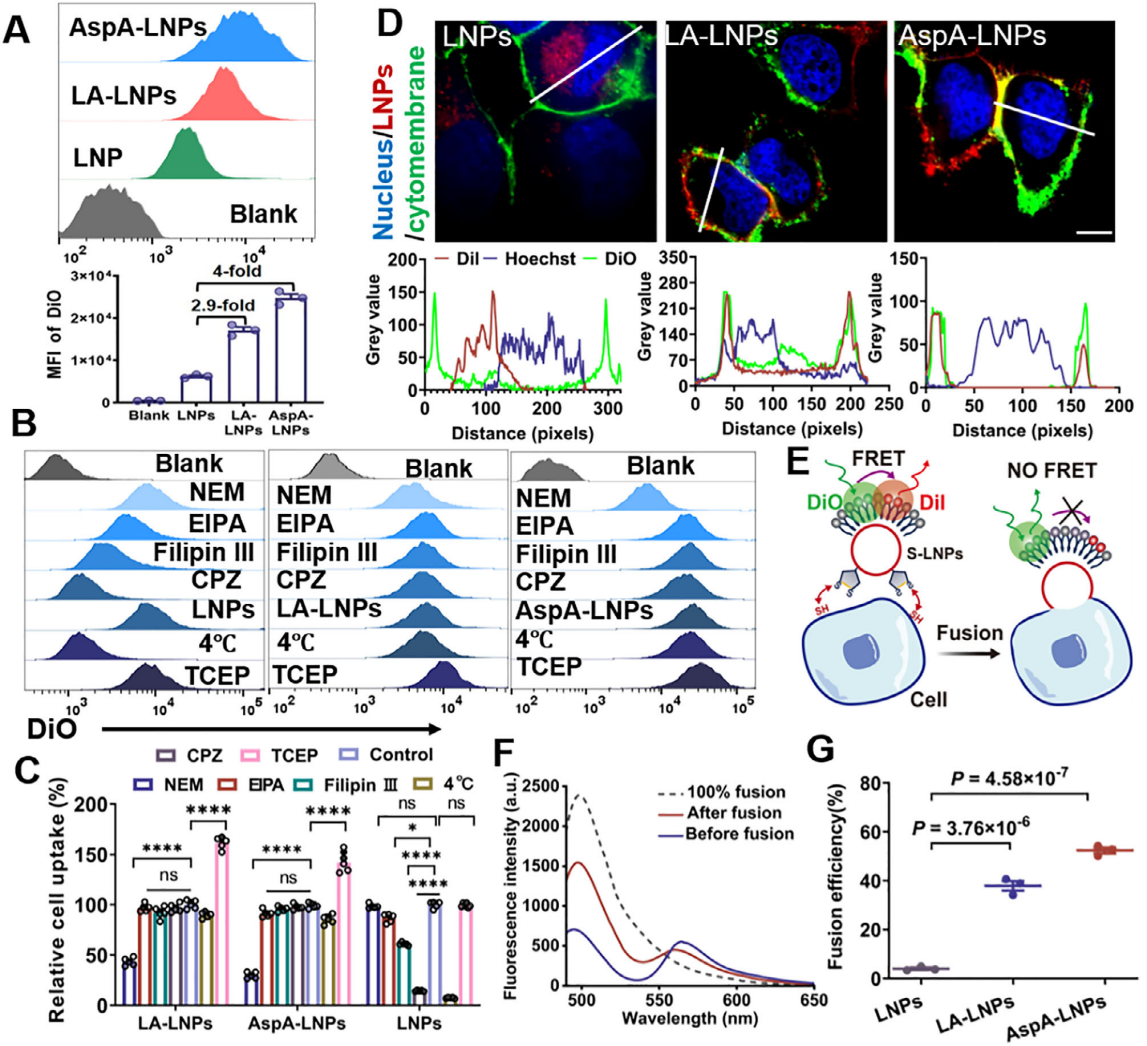
Evaluating the cellular uptake pathway of thiolated LNPs. (A) Flow cytometry analysis of HeLa cells incubated with DiO‐labeled LNPs, LA‐LNPs, and AspA‐LNPs (n = 3). (B) Representative flow cytometry plots of HeLa cells pretreated with different inhibitors for 30 min, followed by co‐incubation with LNPs, LA‐LNPs, and AspA‐LNPs for 2 h, respectively. (C) Quantification of the relative cellular uptake of LNPs under the indicated treatment conditions. The control group denotes the corresponding LNPs, LA‐LNPs, and AspA‐LNPs, respectively, and their cellular uptake is normalized to 100% (n = 5). (D) CLSM images of DiO‐labeled cell membrane (green), DiI‐labeled LNPs (red), and Hoechst 33342‐labeled nuclei (blue). Scale bar, 10 µm. (E) Schematic illustration of the FRET assay for monitoring membrane fusion between HeLa cells and DiO/DiI‐labeled AspA‐LNPs. (F) Fusion changes the fluorescent intensity of donor DiO (501 nm) and acceptor DiI (565 nm). 100% fusion is defined as the complete disruption of LNP structure by 0.1% Triton X‐100 to achieve maximum fusion. (G) The fusion efficiency of LNPs, LA‐LNPs, and AspA‐LNPs with HeLa cell membrane (n = 3). Data are presented as mean ± SD. Statistical significance was determined using ordinary one‐way ANOVA with multiple comparisons in C and G (ns, not significant; ^*^ *p* < 0.05; ^****^ *p* < 0.0001).

We finally assessed the functional consequences of the LA‐ and AspA‐thiolated LNPs and MSNs for miRNA delivery. The miR‐34a‐5p mimic exerts tumor suppressive effects by repressing multiple targets including BCL2, SIRT1, and MET, resulting in the decrease of plasma membrane stiffness [34]. The miR‐34a‐5p mimic was loaded into AspA‐LNPs, AspA‐MSNs, LA‐LNPs, and LA‐MSNs to treat HeLa cells, respectively, and the changes in plasma membrane stiffness were monitored using AFM‐based nanoindentation (Figure S32A). With increasing treatment time, the Young's modulus of cells gradually decreased, and the AspA‐LNPs group exhibited the most pronounced reduction in cell stiffness (Figure S32B,C). CCK‐8 assay demonstrated that the AspA‐LNPs group exhibited greater inhibition of proliferation than the other groups (Figure S33). Quantitative apoptosis analysis revealed that HeLa cells treated with the AspA‐LNPs group exhibited a higher apoptotic fraction (Figure S34). In summary, the superior therapeutic performance of the AspA‐LNPs group derives from the effect of highly reactive cyclic disulfide and soft LNP carriers during transmembrane transport.

## 3 Conclusion

In summary, we revealed the effect of ligand reactivity and carrier rigidity on the cellular uptake of thiolated miRNA nano‐drugs. The interaction dynamics for the cyclic disulfide exchanging with thiols on TFRC of the cell membrane were analyzed at the single‐molecule level. Compared with LA, AspA exhibits higher binding affinity, greater binding stability, and more double‐disulfide binding, as well as greater membrane perturbation and deeper insertion capability. However, the concentration‐dependent self‐activation and self‐inhibition effects of LA and AspA on reacting with thiols were observed, indicating that their binding is regulated by collective nanoscale interactions. AspA induces denser and more cooperative receptor clustering and displays greater enrichment at TFRC sites. The thiolated rigid MSNs tend to enter cells through receptor‐mediated endocytosis; thiolation enhances cellular uptake efficiency by reducing the force and duration required for miRNA nano‐drug internalization and significantly accelerating transmembrane speed. Whereas the thiolated soft LNPs tend to entry cell via membrane fusion, the AspA thiolated LNPs showed higher efficiency of membrane fusion, which is likely facilitated by the combined action of enhanced thiol‐exchange‐mediated membrane anchoring and deeper lipid insertion. Functionally, AspA‐LNPs loaded with miR‐34a‐5p mimic yield the higher apoptotic response in HeLa cells with larger reductions in membrane stiffness. These results will define validated design principles for thiol‐mediated nano‐drug delivery, and these principles could also be extended to additional ligand chemistries and carrier architectures for nano‐drug delivery systems.

## 4 Experimental Section

### 4.1 Materials

Octanoic acid, 8‐[(2‐hydroxyethyl)[6‐oxo‐6‐(undecyloxy)hexyl]amino]‐, 1‐octylnonyl ester (SM‐102; Cat. No.S1507326), 1,2‐distearoyl‐sn‐glycero‐3‐phosphocholine (DSPC; Cat. No. D1520096), and Cholesterol (Chol; Cat. No. C104036) were purchased from Shanghai Aladdin Biochemical Tech Co., Ltd. 1,2‐distearoyl‐sn‐glycero‐3‐phosphoethanolamine‐*N*‐[amino(polyethylene glycol)‐2000] (DSPE‐PEG_2000_‐NH_2_; Cat. No. CP0204‐1 g) and azido‐poly(ethylene glycol)_400_‐amine (N_3_‐PEG_400_‐NH_2_; Cat. No. CP0157‐1 g) were purchased from Guangzhou Weishi Optical Techno Co., Ltd. Asparagusic acid (AspA; Cat. No. S84495) and lipoic acid‐N‐hydroxysuccinimide ester (LA‐NHS; Cat. No. Y57034) were purchased from Shanghai yuanye Bio‐Technology Co., Ltd. micrON mimic NC #22 (Cat. No. miR1N0000002‐1‐10) and micrON mimic NC #22 (5Cy3) (Cat. No. miR01102‐1‐1) were purchased from Suzhou Ruibo Biotechnology Co., Ltd. The T7 peptide (KHAIYPRH) was custom‐synthesized by GenScript Biotech Corporation.

### 4.2 Cell Culture

HeLa cells were purchased from the Shanghai Academy of Biological Sciences (Shanghai, China; Cat. No. CL‐0101; RRID: CVCL 0030). HeLa cells were separately cultured in Dulbecco's Modified Eagle's Medium (DMEM). The DMEM and MEM were supplemented with 10% fetal bovine serum (FBS), 1% streptomycin, 1% penicillin, and BIOMYC‐3 antibiotic. The cells were incubated in a constant‐temperature incubator maintained at 37°C with 5% CO_2_.

### 4.3 Preparation of SPD‐1 LNPs and SPD‐2 LNPs

Thiolated LNPs were prepared by the thin‐film hydration method. Precisely calculated volumes of SM‐102, Chol, DSPC, thiolated lipid, and DSPE‐PEG_2000_ were dissolved in chloroform at various molar ratios, as detailed in Table S2, yielding a total lipid concentration of 10 mM. The solvent was removed using rotary evaporation to form a thin lipid film, which was further dried under a nitrogen stream to eliminate residual solvent. The film was hydrated with 1 mM 4‐(2‐hydroxyethyl)‐1‐piperazineethanesulfonic acid (HEPES, pH 7.4) buffer under constant agitation, followed by sonication using an ultrasonic cell disruptor to generate LNPs. The resulting LNPs were repeatedly extruded through polycarbonate membranes using a mini‐extruder to achieve a uniform size distribution. Extravesicular components were removed via ultrafiltration (30 kDa MWCO, 5000 rpm, 20 min), and the purified LNPs were stored at 4 °C. Cell viability was assessed using the Cell Counting Kit‐8 (CCK‐8) assay (Beyotime Biotechnology, China) to evaluate the cytotoxicity of LNPs.

### 4.4 dSTORM Sample Preparation

Well‐cultured cells were washed three times with warm phosphate‐buffered saline (PBS), post‐fixed with 4% paraformaldehyde (PFA) at RT for 10 min, and washed three times with PBS. The samples were incubated with 3% bovine serum albumin (BSA, w/v) for 30 min to block non‐specific binding sites, and washed three times with PBS to remove the excess BSA. Then, the cells were stained with AspA‐Cy5 or LA‐Cy5 at 4 °C for 10 min in the dark, and washed three times with PBS. Before imaging, the sample was sealed with 100 µL of imaging buffer containing 0.5 mg mL^−1^ glucose oxidase (100000 U/g) and 40 mg mL^−1^ catalase.

### 4.5 dSTORM Imaging and Data Analysis

dSTORM imaging was performed using an HM‐SR‐01 STORM microscope (Microfield Optics, Changchu). Fluorescent excitation was achieved using a 640 nm laser or 532 nm laser, while a low‐power 405 nm laser (0.5 mW) was employed to enhance the number of fluorophores in the on‐state. During imaging, we used two clips to minimize *x*‐*y* drift and an autofocus lock to eliminate z‐drift. Continuous capture of 5000 images per cell with a 24 ms exposure time was facilitated by Micro‐Manager software. The raw data were analyzed using ThunderSTORM, a freely available ImageJ plug‐in, to generate the reconstructed dSTORM images [35]. Single‐molecule localization datasets of membrane thiols were analyzed using SR‐Tesseler. In this method, the super‐resolution image was subdivided into a number of polygons centered on localizations. Once the region of interest (ROI) was circled and suitable thresholds were set, polygons were created within the ROI to outline objects.

### 4.6 AFM Tip Modification

The AFM tips (MSCT, D‐tip with a normal spring constant of 0.03 N/m, Bruker, USA) were cleaned using piranha solution (98% H_2_SO_4_: 30% H_2_O_2_ = 7:3, v/v) and ozone conditions successively. The spring constant of the AFM tip cantilever was calibrated by the thermal noise method. Subsequently, the AFM tip was incubated by vapor deposition with 50 µL of 3‐aminopropyltriethoxysilane (APTES) and 20 µL of N, N‐diisopropylethylamine. The silylated AFM tip was combined with heterobifunctional PEG linkers (Acetal‐PEG‐NHS, 1 mg mL^−1^) in trichloromethane containing 0.5% triethylamine (v/v) for 2 h. The PEG‐modified AFM tip was immersed in 1% citric acid for 10 min to activate the carboxyl aldehyde. The AFM tip was then immersed in a mixture of thiol‐MSNs and 1 M NaCNBH_3_ for 2 h. Finally, 10 µL of 1 M ethanolamine was added to the reaction solution for 15 min to deactivate the unreacted aldehyde groups. The Fmoc‐NH‐PEG‐NHS linker was used to connect the silylated AFM tip and LA/AspA. The silylated AFM tip was combined with the Fmoc‐NH‐PEG‐NHS (1 mg mL^−1^) in a toluene solution containing 0.5% triethylamine (v/v) for 2 h. To remove the protecting group of 9‐fluorenylmethoxycarbonyl (Fmoc) and obtain an amino group, the PEG‐modified AFM tip was immersed in a 20% 4‐methylpiperidine (4‐MP) solution for 20 min. The AFM tip was then immersed in an LA/AspA solution for 2 h. The functionalized AFM tips were rinsed with PBS and immediately used for experiments.

### 4.7 SMFS Measurements

SMFS measurements were performed using an AFM 5500 (Agilent Technologies, USA). The interaction between LA/AspA and thiol groups on the cell membrane was measured in contact mode. The dwell time was determined from the force‐distance (FD) curve, i.e., the time needed to travel from the contact point to the maximum force limit (relative maximum deflection limit of 0.2 V) and back again. According to the single‐barrier model, the correlation between rupture force and loading rate is rationalized by the approximate form of the equation [36]:

(1) $$F_{r}=\frac{k_{B}T}{X_{\beta }}\;\ln\left(\frac{X_{\beta }}{k_{\mathrm{off}}^{0}k_{B}T}r\right)$$

where *F*_r_ is the rupture force, *X*_β_ is the separation distance from the equilibrium position, *r* is the loading rate, *k*_off_ is the dissociation kinetic rate constant at zero force, *k*_B_ is the Boltzmann constant, and *T* is the Kelvin temperature. The dissociation activation energy *ΔG*_β,0_ in the absence of an external force is evaluated as:

(2) $$\Delta G_{\beta ,0}=-k_{B}\mathrm{Tln}\left(\tau_{D}k_{\mathrm{off}}\left(0\right)\right)$$

where τ_D_ denotes the diffusive relaxation time; we used τ_D_ = 10^−9^ s. It is assumed that the receptor‐bond complex could be approximated by pseudo‐first‐order kinetics. *k*_on_ for LA/AspA binding to membrane thiols was extracted from the binding probability (*BP*) measured at varying contact time [37]:

(3) $$k_{on}=\frac{\frac{1}{2}\cdot 4\pi r_{eff}^{3}\cdot N_{A}}{3n_{b}\tau }$$

where *r*_eff_ is the effective radius of the sphere, which is the sum of the PEG linker length (3.22 nm) and the dithiolane diameter (LA and AspA were respectively 0.02 and 0.01 nm). *n_b_* is the number of bound ligands, while *N_A_* is the Avogadro constant. *τ* is the interaction time, which can be calculated from the binding probability at different dwell times using the following equation:

(4) $$BP=A^{*}\;\left[1-\exp\left(\frac{-\left(t-t_{0}\right)}{\tau }\right)\right]$$

where *A* is the maximum *BP*, *t_0_* is the lag time, and *t* is a certain holding time when the AFM tip contacts the sample surface. The *BP* was measured at 0.16, 0.32, 0.64, 0.96, and 1.44 s. Origin software was used to fit the data and extract the *τ*.

### 4.8 Molecular Dynamics Simulations

A bilayer containing 200 POPC molecules (100 per leaflet) was constructed using CHARMM‐GUI Membrane Builder, then solvated with water containing 0.15 M NaCl and neutralized as needed [38]. In each system, the LA or AspA was initially placed in the aqueous phase approximately 1.5 nm from the membrane plane. Nonbonded parameters and AM1‐BCC charges for the small molecules were generated with antechamber using the GAFF force field and converted to GROMACS topology files using ParmEd and ACPYPE for compatibility with the CHARMM36 lipid force field. All MD simulations were performed with GPU acceleration in GROMACS v2020.6. Systems underwent 5,000 steps of energy minimization and were heated to 310 K over 2 ns, and equilibrated for 10 ns with positional restraints gradually released. Production runs were performed in triplicate for 100 ns under NPT conditions with a 2 fs timestep, employing a Nose‐Hoover thermostat at 310 K and semi‐isotropic Parrinello‐Rahman pressure coupling at 1 bar. Long‐range electrostatics were treated with the PME method and nonbonded interactions used a 1.0 nm cutoff. To probe the effect of surface tension on membrane‐small molecule interactions, a constant surface tension of 40 dyne cm^−1^ (0.04 N m^−1^) was applied in the x and y plane during production.

### 4.9 Force Tracing Measurements

Prior to force tracing measurements, 2 mL of DMEM was added to the cell culture dish. All force tracing experiments were performed in DMEM without FBS to avoid interference from macromolecular components in FBS. The engulfment depth (*D*) is equal to the sum of the bending distance (*d*) of the AFM tip cantilever and the stretching length (*h*) of the PEG linker, as shown in the formula below:

(5) D=d+h

The bending distance (*d*) of the AFM tip cantilever was calculated from Hooke's law:

(6) F=k×d

where *F* is the endocytic force for single nanoparticle entry into the cell, and *k* is the spring constant of the AFM tip cantilever. Because the force (*F*)‐dependent stretching behavior of PEG can be described by the extended worm‐like chain (WLC) model, the stretching length (*h*) of the PEG linker was calculated according to the following formula:

(7) $$\frac{FL_{p}}{k_{B}T}=\frac{1}{4}{\left(1-{\frac{h}{L}}_{0}+\frac{F}{k_{0}}\right)}^{-2}\;-\frac{1}{4}+{\frac{h}{L}}_{0}-\frac{F}{k_{0}}$$

where *k*_B_ is the Boltzmann constant, *T* is the absolute temperature, *L*p is the persistence length, the enthalpic correction *k*_0_ is 1561±33 pN, and *L*_0_ is the contour length. As previously reported, the PEG unit length is 4 Å and the terminus is 5 Å; the estimated total contour length of the PEG_3400_ linker is around 322 Å.

### 4.10 Statistical Analysis

Statistical analyses were performed using GraphPad Prism 10 (GraphPad Software, San Diego, CA, USA). Data are presented as individual data points with mean ± standard deviation (SD) unless otherwise indicated. Statistical tests were selected based on the experimental design, data distribution, and number of groups. One‐way analysis of variance (ANOVA) was used to compare three or more groups. When ANOVA indicated statistical significance, appropriate post hoc tests were used depending on the comparison strategy. Dunnett's multiple comparison test was used for comparisons relative to the single control group. Tukey's multiple comparison test was used for pairwise comparisons among groups. Fisher's least significant difference (LSD) test was used for predefined comparisons between selected groups. For direct comparisons between the two groups, an unpaired two‐tailed Student's *t*‐test was performed. For datasets that did not meet the assumptions of normality or homogeneity of variance, the non‐parametric Kruskal–Wallis test followed by Dunn's multiple‐comparisons test was applied. Statistical significance was defined as follows: ^*^ *p* < 0.05, ^**^ *p* < 0.01, ^***^ *p* < 0.001, ^****^ *p* < 0.0001, and ns, not significant.

## Author Contributions

**Zhuang Zhang**: Conceptualization, Methodology, Data Curation, Visualization, Writing – Original Draft, Writing – Review & Editing. **Hui Wang**: Data Curation. **Hao Zhang**: Data Curation. **Zhenkuan Cao**: Data Curation, Software. **Zehui Liu**: Data Curation, Formal Analysis. **Hongda Wang**: Conceptualization, Methodology, Writing – Original Draft, Writing – Review & Editing. **Siying Li**: Methodology, Writing – Original Draft, Writing – Review & Editing. **Yuping Shan**: Methodology, Resources, Funding Acquisition, Writing – Original Draft, Writing – Review & Editing. All authors reviewed and approved the final version of the manuscript.

## Conflicts of Interest

The authors declare no conflicts of interest.

## Supporting information




**Supporting File**: advs77389‐sup‐0001‐SuppMat.docx.

## Data Availability

The data that support the findings of this study are available from the corresponding authors upon request.
