# Supplementary material for: The Effect of Ligand Reactivity and Carrier Mechanics on Thiol‐Mediated Cellular Uptake for miRNA Nano‐Drugs
Source: Adv Sci (Weinh). 2026 Aug 27:e77389. Online ahead of print. doi: 10.1002/advs.77389 (PMC13521186; doi:10.1002/advs.77389)
Supplement: Supplementary file 1 [file ADVS-9999-e77389-s001.docx]

**Supporting Information**

**The Effect of Ligand Reactivity and Carrier Mechanics on Thiol-Mediated Cellular Uptake for miRNA Nano-Drugs**

Zhuang Zhang^a^, Hui Wang^a^, Hao Zhang^a^, Zhenkuan Cao ^a^, Zehui Liu^a^, Siying Li^a,^ *, Hongda Wang^b,^ *, Yuping Shan^a,^ *

^a^*College of Chemistry and Life Science, Advanced Institute of Materials Science, Changchun University of Technology, Changchun 130012, China*

^b^*State Key Laboratory of Electroanalytical Chemistry, Changchun Institute of Applied Chemistry, Chinese Academy of Sciences, Changchun 130022, China*

*Corresponding authors. E-mail: [shanyp@ciac.ac.cn](mailto:shanyp@ciac.ac.cn); lisiying@ccut.edu.cn; hdwang@ciac.ac.cn

***KEYWORDS****: Disulfide-thiol exchanging kinetics • Force tracing • Single molecule force spectroscopy • Super-resolution fluorescence imaging • Thiol-mediated uptake dynamics*

**Table of Contents**

[**Experimental section**	3](#_Toc236316261)

[**Figure S1.**	5](#_Toc236316262)

[**Table S1.**	5](#_Toc236316263)

[**Figure S2.**	5](#_Toc236316264)

[**Figure S3.**	6](#_Toc236316265)

[**Figure S4.**	6](#_Toc236316266)

[**Figure S5.**	7](#_Toc236316267)

[**Figure S6.**	7](#_Toc236316268)

[**Figure S7.**	7](#_Toc236316269)

[**Figure S8.**	8](#_Toc236316270)

[**Figure S9.**	8](#_Toc236316271)

[**Figure S10.**	8](#_Toc236316272)

[**Figure S11.**	8](#_Toc236316273)

[**Figure S12.**	9](#_Toc236316274)

[**Figure S13.**	9](#_Toc236316275)

[**Table S2.**	10](#_Toc236316276)

[**Figure S14.**	10](#_Toc236316277)

[**Figure S15.**	10](#_Toc236316278)

[**Figure S16.**	11](#_Toc236316279)

[**Figure S17.**	11](#_Toc236316280)

[**Figure S18.**	11](#_Toc236316281)

[**Figure S19.**	12](#_Toc236316282)

[**Figure S20.**	12](#_Toc236316283)

[**Figure S21.**	13](#_Toc236316284)

[**Figure S22.**	13](#_Toc236316285)

[**Figure S23.**	13](#_Toc236316286)

[**Figure S24.**	14](#_Toc236316287)

[**Figure S25.**	14](#_Toc236316288)

[**Figure S26.**	14](#_Toc236316289)

[**Figure S27.**	15](#_Toc236316290)

[**Figure S28.**	16](#_Toc236316291)

[**Figure S29.**	16](#_Toc236316292)

[**Figure S30.**	16](#_Toc236316293)

[**Figure S31.**	17](#_Toc236316294)

[**Figure S32.**	17](#_Toc236316295)

[**Figure S33.**	18](#_Toc236316296)

[**Figure S34.**	18](#_Toc236316297)

**Experimental section**

**Stability of thiol-functionalized LNPs and MSNs**

The thiol-functionalized LNPs were freshly prepared and incubated with 10% (v/v) FBS, respectively. Then the sizes and PDI of LA-LNPs and AspA-LNPs were investigated by dynamic light scattering (DLS, Malvern Zetasizer) in PBS at different time points (7, 14, 28, 35, or 45 days).

**Membrane fusion assay**

HeLa cells were seeded at 5 × 10^4^ cells per 35-mm confocal dish (NEST Biotechnology Co., Ltd., China) and incubated for 24 h. Cell membranes were labeled with DiO (final concentration 5 µM) for 10 min, followed by gentle washes with pre-warmed PBS to remove unincorporated dye. DiI-labeled LNPs were added to the dish and incubated for 2 h. Samples were fixed with 4% PFA and nuclei counterstained with Hoechst 33342. Confocal images were acquired using an Andor (Oxford Instruments) spinning disk confocal imaging microscope (CLSM) and subsequently analyzed.

LNPs were dual-labeled with DiO and DiI to enable assessment of membrane fusion by Förster resonance energy transfer (FRET). When these LNPs fused with cell membranes, the increased donor-acceptor distance significantly reduced FRET efficiency. The baseline of FRET intensity (*F_0_*) was measured (excitation 483 nm, emission 565 nm), and complete disruption (*F_100_*) was achieved by adding 0.1% (v/v) Triton X-100. After incubation with cells at different time intervals, the fluorescence intensity (*F_DiO_*) of LNPs/cell mixtures was recorded to determine membrane fusion performance using the formula:

$\text{Fusion(\%)=(}\text{F}_{\text{DiO}}\text{-}F_{\text{0}}\text{)/(}\text{F}_{\text{100}}\text{-}\text{F}_{0}\text{)}\leftarrow$(1)

**Flow cytometry analysis**

HeLa cells were incubated with thiolated LNPs or thiolated MSNs for 1 h and then trypsinized for 3 min. Cells were collected by centrifugation at 1000 rpm for 5 min at 4 °C, and resuspended in 500 µL of cold PBS. The cells were then analyzed using a BD FACS Accuri™ C6 flow cytometer, with 10,000 events acquired per sample. Data were analyzed using FlowJo V10 software.

**Cellular uptake inhibition assay**

To elucidate the endocytic pathway underlying the cellular internalization of thiol-modified LNPs and MSNs, HeLa cells were subjected to pharmacological inhibition under controlled conditions. Specifically, cells were preincubated at 37 °C for 30 min with N-ethylmaleimide (NEM, 500 nM), chlorpromazine (CPZ, 10 µM), Filipin III (100 nM), or 5-(N-ethyl-N-isopropyl) amiloride (EIPA, 50 nM). After pretreatment, thiol-modified LNPs or MSNs were incubated with cells for 1 h. Unbound nanoparticles were washed away using PBS, followed by replacement with fresh DMEM for subsequent analyses, including force tracing, flow cytometry, and confocal microscopy.

**TFRC knockdown and cellular uptake analysis**

HeLa cells were transfected with TFRC-targeting siRNA or negative-control siRNA according to the manufacturer’s instructions. After transfection for 48 h, TFRC expression was measured by flow cytometry, and the expression level in the control group was normalized to 100%. For the uptake assay, control and TFRC siRNA-transfected cells were incubated with Cy5-labeled MSNs, LA-MSNs, or AspA-MSNs for 1 h. The cells were then washed, harvested, and analyzed by flow cytometry. Nanoparticle uptake was quantified using the mean fluorescence intensity. Data were obtained from three independent experiments and are presented as the mean ± SD (n = 3).

**Confocal FRAP analysis of supported lipid bilayers**

Supported lipid bilayers (SLBs) composed of DPPTE and DOPC at a molar ratio of 5:95 was fluorescently labeled with DiO. The SLBs were treated with LNPs, LA-LNPs, or AspA-LNPs, while untreated SLBs served as the control. The fluorescence recovery after photobleaching (FRAP) measurements were performed using a confocal microscope with a 20 × 20 μm square bleaching region. Fluorescence images were acquired before bleaching, immediately after bleaching, and during fluorescence recovery. The post-bleaching fluorescence intensity profile was fitted to determine the effective bleaching radius. Recovery curves were background-corrected and normalized to calculate the effective diffusion coefficient and mobile fraction. Ten regions of interest from three independent experiments were analyzed for each group.

**Preparation of schematic illustrations**

The three-dimensional models and rendered elements used in the schematic illustrations and the Table-of-Contents graphic were created using Cinema 4D R19 (Maxon Computer GmbH, Germany). Graphical assembly, annotation, and final layout were performed using Adobe Illustrator 2021 (Adobe Inc., USA).

Figure S1. LA and AspA were attached to the AFM tip *via* a heterobifunctional PEG linker.
Table S1. Kinetic parameters characterizing the interaction between membrane thiols and LA/AspA.

|  | ***τ* (s)** | ***k*_on_ (M^-1^s^-1^)** | ***k*_off_ (s^-1^)** | ***K*_a_ (M^-1^)** | ***X*_β_ (nm)** | *Δ***G_β,0_ (k_B_T)** |
| --- | --- | --- | --- | --- | --- | --- |
| LA | 0.29 | 1.52×10^3^ | 6.3×10^-1^ | 2.41×10^3^ | 0.34 | 21.19 |
| AspA | 0.21 | 2.09×10^3^ | 4.0×10^-3^ | 5.22×10^5^ | 0.21 | 26.24 |

Figure S2. Characterization of the TFRC-coated surface. (A) AFM topography image of the TFRC-coated surface after scanning of a 1 µm^2^ area in the center with a high applied force (~25 nN) to remove attached molecules, scale bar 500 nm. (B) The corresponding height profile reveals an ~4.1 nm depression in the cleared region.

Figure S3. SMFS of LA/AspA interacting with TFRC. (A) Typical FD curves for the interaction between LA/AspA and TFRC. The black arrows represent the rupture force signals. (B, C) PDFs plot indicating the distribution of the rupture force between TFRC and LA (B) or AspA (C). The data were fitted with a bimodal Gaussian model. (D) The BP of LA/AspA binding to TFRC (n=5).

Figure S4. Schematic illustration of AspA/LA binding to membrane thiols based on the Bell-Evans two-state model. The transition from the bound state to the unbound state is separated by an energy barrier located at distance *X*_β_. *k*_off_ indicates the dissociation rate observed with no external force (F = 0) or an external force (F > 0) applied onto the bond.

Figure S5. Comparison of the potentials of mean force for AspA and LA along the POPC membrane-normal reaction coordinate. Umbrella-sampling PMFs interpolated onto a common 0.00-3.50 nm grid. Each profile was independently referenced to the mean energy in bulk water at 3.3-3.5 nm. Solid lines show the full-data PMFs, shaded regions indicate the 2.5th-97.5th percentiles of 200 bootstrap profiles, and circles mark the full-profile minima.

Figure S6. UV-Vis Spectra characterization of AspA-FITC, LA-FITC, AspA-Cy5, LA-Cy5 and T7-Cy3.

Figure S7. dSTORM images of membrane thiols bound with LA or AspA at different concentration, scale bar: 3 μm.

Figure S8. ^1^H NMR spectrum of DSPE-PEG_2000_-NH_2_ in CDCl_3_.

Figure S9. ^1^H NMR spectrum of SPD-1 in CDCl_3_.

Figure S10. ^1^H NMR spectrum of SPD-2 in CDCl_3_.

Figure S11. Representative energy dispersive spectroscopy profile of SPD-1 and SPD-2.

Figure S12. Quantification of SPD-1 and SPD-2 yields using the ninhydrin assay. (A) UV-Vis spectra of the amino groups in the standard amino acids at different concentration. (B) Calibration curve used for quantifying the synthesis yield.

Figure S13. Quantification of SPD-1 and SPD-2 in LNPs using the thiol-reactive fluorescent dye Cy3-SH. (A) UV-Vis spectra of Cy3-SH at different concentration. (B) Calibration curve used for quantifying the thiolated lipid concentration in LNPs.
Table S2. Detailed formulation of LNPs with varying concentration of thiolated lipids. Lipid components are presented as corresponding input molar ratios.

| **molar ratios (%)** | | | | | | |
| --- | --- | --- | --- | --- | --- | --- |
| **Name** | **SM-102** | **Chol** | **DSPC** | **DSPE-PEG** | **SPD-1** | **SPD-2** |
| LNP | 50 | 38.5 | 10 | 1.5 | 0 | 0 |
| LA-LNP1 | 50 | 38.5 | 10 | 1 | 0.5 | 0 |
| LA-LNP2 | 50 | 38.5 | 10 | 0.8 | 0.7 | 0 |
| LA-LNP3 | 50 | 38.5 | 10 | 0 | 1.5 | 0 |
| AspA-LNP1 | 50 | 38.5 | 10 | 1 | 0 | 0.5 |
| AspA-LNP2 | 50 | 38.5 | 10 | 0.8 | 0 | 0.7 |
| AspA-LNP3 | 50 | 38.5 | 10 | 0 | 0 | 1.5 |

Figure S14. Quantification of the encapsulation efficiency of miRNA based on the Quant-iT™ RiboGreen^®^ RNA Assay. (A) Emission spectrum of miRNA at different concentration. (B) Corresponding standard curve used for data analysis.

Figure S15. Cytotoxicity of thiolated LNPs and MSNs formulations at varying concentration (n = 5).

Figure S16. Representative FTIR spectroscopy profile of MSNs, LA-MSNs, and AspA-MSNs.

Figure S17. dSTORM images of miRNA-loaded thiolated MSNs. Scale bar, 1 µm.

Figure S18. dSTORM super-resolution imaging analysis of miRNA-loaded LNPs, LA-LNPs, and AspA-LNPs. (A) Single-molecule localization maps representing the spatial distribution of individual emitters. (B) Histogram distribution of single-molecule localization precision (sigma values). The red line represents the best-fit normal distribution (Gaussian function). (C, D) Gaussian fitting of the spatial distribution along the x (C) axes and y (D) axes.

Figure S19. dSTORM super-resolution imaging analysis of miRNA-loaded MSNs, LA-MSNs, and AspA-MSNs. (A) Single-molecule localization maps representing the spatial distribution of individual emitters. (B) Histogram showing the frequency distribution of single-molecule localization precision (sigma values). The red line represents the best-fit normal distribution (Gaussian function). (C, D) Gaussian fitting of the spatial distribution along the x (C) axes and y (D) axes.

Figure S20. Schematic of thiolated MSNs modified AFM tip. (A) LA-MSNs, (B) AspA-MSNs and (C) MSNs were attached to the AFM tip via a heterobifunctional PEG linker, respectively.

Figure S21. The optical image of the AFM tip cantilever locating above the living HeLa cells, the red dot indicates the position of AFM tip. Scale bar: 50 μm.

Figure S22. Determining the contact point between the thiolated MSNs modified AFM tip and the cell surface. The contact point is the intersection of the slope and the flat part in the force-distance curve, indicating by the red arrow.

Figure S23. Schematic diagram for monitoring the cellular uptake of a single AspA-MSN by force tracing.

Figure S24. Probability of observing FT signal for MSNs entry cell (n = 10).

Figure S25. Analysis of typical noise level and signal-to-noise ratio in force-time curves from force tracing experiments. (A) Baseline noise of a FT curve. (B) Signal-to-noise ratio of a FT curve.

Figure S26. Calculating the displacement during the process of MSN, AspA-MSN and LA-MSN entry cell. (A) Schematic of the displacement during the process of AspA-MSN entry cell, consisting the bending distance of the AFM tip cantilever (d) and the extension length of the PEG linker (h). (B) The box plot statistics analysis of the displacement. The boxes mean the main distribution (50%) of values, the vertical lines through boxes represent the 5% to 95% data range, the horizontal line in every box represents the median, and the square in each box represents the mean value. The data were normally distributed. The *P* value was compared using unpaired two-tailed Student's *t*-test (ns, no significance).

Figure S27. Effect of TFRC knockdown on the cellular uptake of MSNs, LA-MSNs, and AspA-MSNs in HeLa cells. (A) Representative flow cytometry plots of TFRC expression in control and TFRC siRNA-transfected HeLa cells. (B) Quantification of relative TFRC expression and TFRC knockdown efficiency. TFRC expression in the control group was normalized to 100%. (C) Representative flow cytometry plots of control or TFRC siRNA-transfected HeLa cells following co-incubation with MSNs, LA-MSNs, or AspA-MSNs for 1 h. (D) Quantification of the mean fluorescence intensity (MFI) of MSNs, LA-MSNs, and AspA-MSNs in control and TFRC siRNA-transfected HeLa cells. Data are presented as the mean ± SD from three independent experiments (n = 3). Statistical significance was determined using ordinary one-way ANOVA with multiple comparison (ns, not significant; *****P* < 0.0001).

Figure S28. Quantification of cell surface thiols on HeLa cells using the DTNB assay.

Figure S29. FRET-based fluorescence changes of DiO (donor) and DiI (acceptor) upon membrane fusion at 484 nm excitation, characterized by decreased acceptor emission and enhanced donor emission. (A, B) Corresponding emission spectra under the DiO/DiI-labeled LA-LNPs and DiO/DiI-labeled LNPs interacting with HeLa cells.

Figure S30. Confocal fluorescence images of HeLa cells treated with LNPs, LA-LNPs, or AspA-LNPs. miRNA was labeled with Cy3 (red), LNPs were labeled with DiD (green), and cell nuclei were stained with DAPI (blue). Representative fluorescence images and corresponding regions of interest (ROIs), as well as magnified views of the selected ROIs, are shown for each group. Scale bars: 10 μm (top) and 5 μm (bottom).

Figure S31. Confocal FRAP analysis of a thiol-containing supported lipid bilayer. The SLB composed of DPPTE and DOPC at a molar ratio of 5:95 was fluorescently labeled with DiO, and FRAP measurements were performed using a square bleaching region with a side length of 20 μm. (A) Representative FRAP images of the DPPTE-SLB after treatment with AspA-LNPs at different time. Scale bar, 10 μm. (B) Raw fluorescence intensity profile across the bleached region of interest (ROI). (C) Effective diffusion coefficient of the DPPTE-SLB in the Control, LNPs, LA-LNPs, and AspA-LNPs groups. (D) Mobile fractions of the DPPTE-SLB in the Control, LNPs, LA-LNPs, and AspA-LNPs groups. Data are presented as the mean ± standard deviation of 10 ROIs obtained from three independent experiments. The *P* value was compared using unpaired two-tailed Student's t-test (*****P* < 0.0001).

Figure S32. Analysis of the cellular uptake efficiency of miRNA-loaded thiolated LNPs and thiolated MSNs. (A) Schematic illustration of nanoindentation performed on cells. (B) Young's modulus of HeLa cells over a 150 min treatment under control and miRNA‑loaded thiolated LNPs or MSNs at 100 nM (n = 6). (C) Interval scatter plot of Young’s modulus of HeLa cells under control and after 150 min treatment with miRNA‑loaded thiolated LNPs or MSNs at 100 nM. Each dot represents that the data from one cell, and bars denote mean ± SD (n = 6). The *P* value was compared using unpaired two-tailed Student's *t*-test (**P* < 0.05; ***P* < 0.01; *****P* < 0.0001).

Figure S33. Cell viability of HeLa cells treated with miRNA-loaded thiolated LNPs and MSNs (n = 5). Data are presented as the mean ± SD. Statistical significance was determined using ordinary one-way ANOVA with multiple comparisons (****P* < 0.001; *****P* < 0.0001).

Figure S34. Flow cytometric analysis of apoptosis in HeLa cells. (A) Representative Annexin V/propidium iodide (PI) flow cytometry plots. (B) Quantification of apoptotic populations (Q2 + Q3 = early and late apoptosis). (n = 3). The *P* value was compared using unpaired two-tailed Student's *t*-test (*****P* < 0.0001).
